# Supplementary material for: Optical and Physicochemical Characterizations of a Cellulosic/CdSe-QDs@S-DAB5 Film
Source: Nanomaterials (Basel). 2022 Jan 29;12(3):484. doi: 10.3390/nano12030484 (PMC8838006; doi:10.3390/nano12030484)
Supplement: Supplementary file 1 [file nanomaterials-12-00484-s001.zip › nanomaterials-1526924-supplementary.pdf]

nanomaterials

Supplementary Information

Optical and Physicochemical Characterizations of a Cellulosic/CdSe-QDs@S-DAB_5 Film

Manuel Algarra [1,*], Ana L. Cuevas [2], Mª Valle Martínez de Yuso [3], Rocío Romero [2], Beatriz Alonso [3], Carmen M. Casado [4] and Juana Benavente [5,*]

[1] INAMAT[2]-Institute for Advanced Materials and Mathematics, Departamento de Ciencias, Universidad Pública de Navarra, Campus de Arrosadía, 31006 Pamplona, Spain
[2] Unidad de Nanotecnología, Centro de Supercomputación y Bioinnovación, Servicios Centrales de Investigación, Universidad de Málaga, 29071 Málaga, Spain; analaura.cuevas@uma.es (A.L.C.); rociorp@uma.es (R.R.)
[3] X-ray Photoelectron Spectroscopy Lab. Central Service to Support Research Building (SCAI), University ofMálaga, 29071 Málaga, Spain; mvyuso@uma.es (M.V.M.d.Y.); beatriz.alonso@uam.es (B.A.)
[4] Departamento de Química Inorgánica, Universidad Autónoma de Madrid, Cantoblanco, 28049 Madrid, Spain; carmenm.casado@uam.es
[5] Departamento de Física Aplicada I, Facultad de Ciencias. Universidad de Málaga, 29071 Málaga, Spain
* Correspondence: manuel.algarra@unavarra.es (M.A.); j_benavente @uma.es (J.B.); Tel.: +34-948-169822 (M.A.)

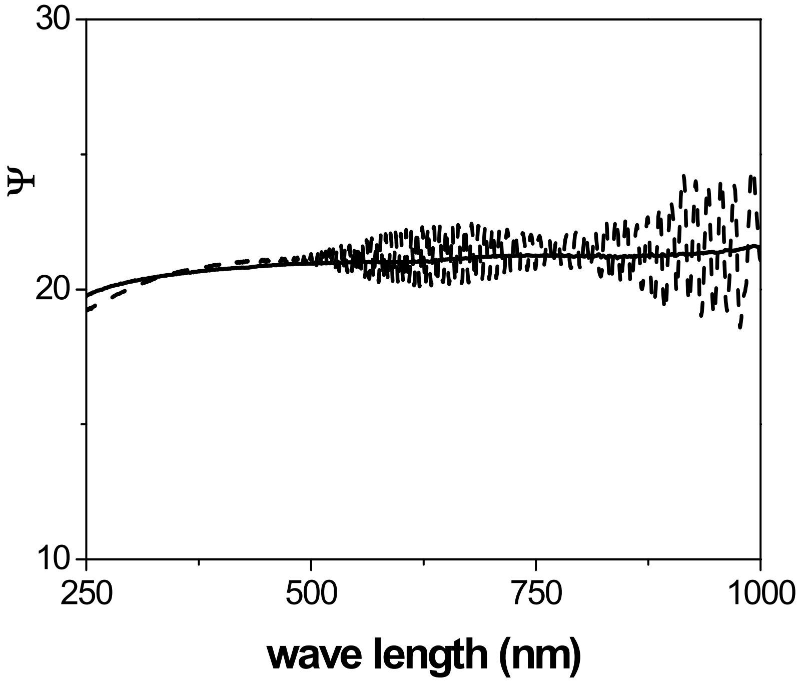

Figure S1. Comparison of wavelength dependence of measured angle (Ψ) for a non-striped RC-4 sample (dashed line) and a striped RC-4 sample (dense line).

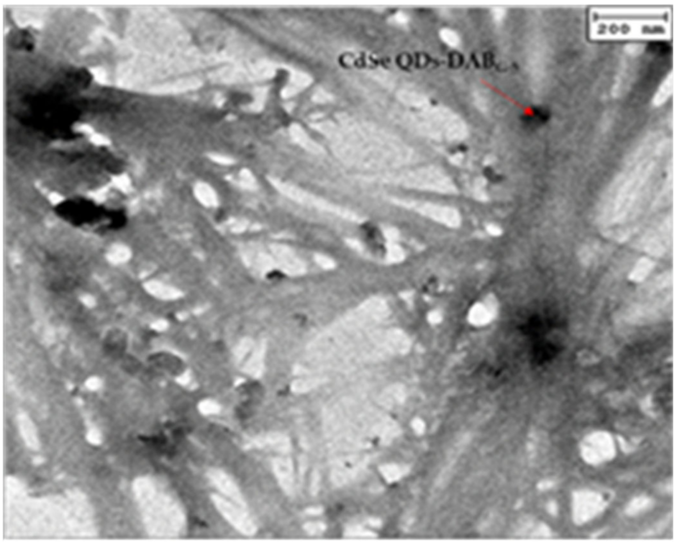

Figure S2. TEM images of CdSe-QDs@S-DAB$_5$ nanoparticles in solution.

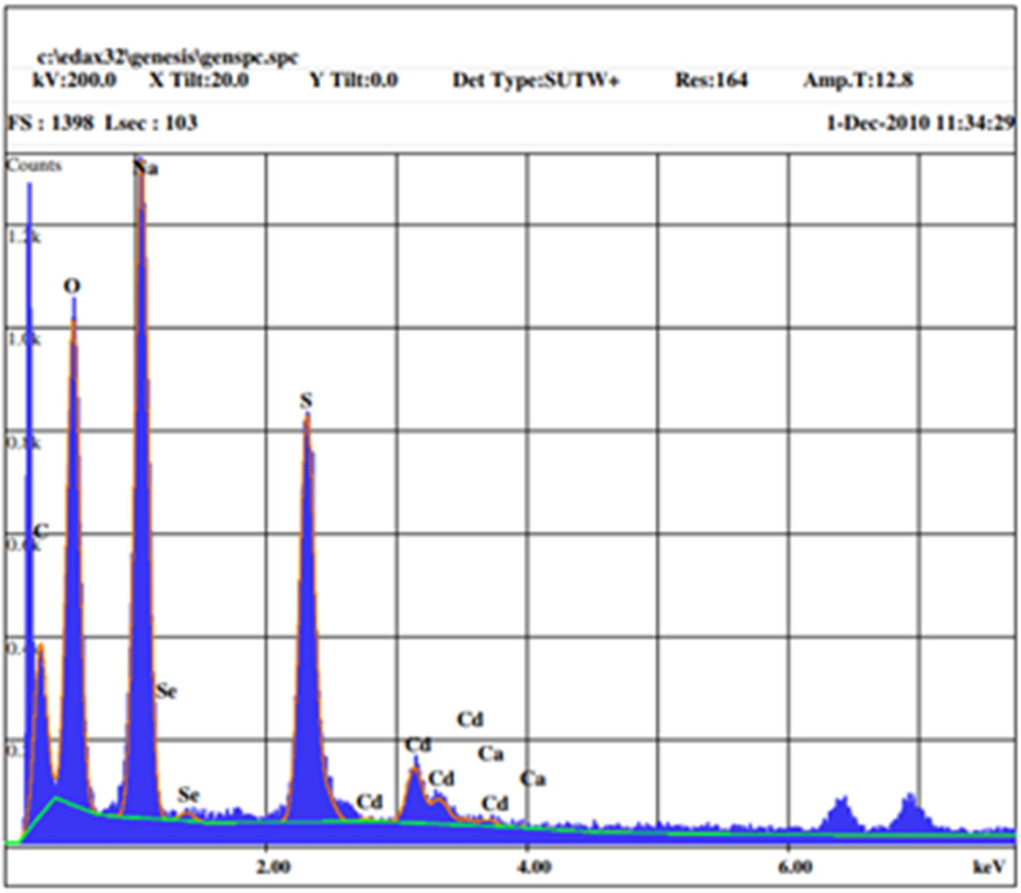

Figure S3. EDAX spectra of the CdSe-QDs@S-DAB$_5$ nanoparticles.

Table S1. Atomic concentration percentages (%) of the elements observed on the surface of the RC-4/CdSe@S-DAB$_5$ film at 70º takes off angle.

Film	C 1*s*	O 1*s*	N 1*s*	Se 3*d*	Cd 3*d*$_5$
**RC-4/CdSe@S-DAB$_5$**	75.6	22.6	0.44	0.03	0.03

Other elements associated to contamination, such as Si, were also detected.
